# Antisocial Personality Disorder, Alcohol, and Aggression

**Published:** 2001

**Authors:** F. Gerard Moeller, Donald M. Dougherty

**Affiliations:** F. Gerard Moeller, M.D., and Donald M. Dougherty, Ph.D., are associate professors in the Department of Psychiatry and Behavioral Sciences, University of Texas—Houston, Houston, Texas

**Keywords:** antisocial personality disorder, aggressive behavior, AODR (alcohol or other drug [AOD] related) behavioral problem, personality trait, AODR violence, expectancy theory of AODU (AOD use, abuse, and dependence), disinhibition theory of AODU, neurobiological theory of AODU, brain function, AODE (effects of AOD use, abuse, and dependence) on emotion

## Abstract

Epidemiologic studies and laboratory research consistently link alcohol use with aggression. Not all people, however, exhibit increased aggression under the influence of alcohol. Recent research suggests that people with antisocial personality disorder (ASPD) may be more prone to alcohol-related aggression than people without ASPD. As a group, people with ASPD have higher rates of alcohol dependence and more alcohol-related problems than people without ASPD. Likewise, in laboratory studies, people with ASPD show greater increases in aggressive behavior after consuming alcohol than people without ASPD. The association between ASPD and alcohol-related aggression may result from biological factors, such as ASPD-related impairments in the functions of certain brain chemicals (e.g., serotonin) or in the activities of higher reasoning, or “executive,” brain regions. Alternatively, the association between ASPD and alcohol-related aggression may stem from some as yet undetermined factor(s) that increase the risk for aggression in general.

Numerous studies indicate an association between alcohol consumption and aggressive behavior. Not all people who consume alcohol, however, become aggressive. In trying to elucidate the relationship between alcohol consumption and aggression, researchers have suggested that people with a psychiatric condition called antisocial personality disorder (ASPD) may be particularly susceptible to alcohol-related aggression. This article explores that association in more detail. First, the article describes the distinguishing features of ASPD. Then it reviews the findings of epidemiologic and laboratory studies that have investigated the link between ASPD and aggression. Finally, the article presents several mechanisms that may contribute to differences between people with and without ASPD with respect to alcohol-related aggression.

## Characteristics of ASPD

According to the *Diagnostic and Statistical Manual of Mental Disorders, Fourth Edition* (DSM–IV) by the American Psychiatric Association (APA) (1994), ASPD is characterized by a pervasive disregard for, and violation of, other people’s rights. The concept of such a personality type is not new. For example, Theophrastus, a student of the ancient Greek philosopher Aristotle, described a personality type that he termed the “unscrupulous man” and which included behaviors that are significant elements of the current concept of ASPD (Millon et al. 1998).

During the past century, researchers and clinicians have used numerous terms to describe ASPD, including “moral insanity,” “psychopathy,” and “sociopathy.” Likewise, the symptoms considered to be the key elements of psychopathy or an antisocial personality have evolved from a focus on the lack of emotional attachment in relationships with others (Cleckley 1964) to a greater focus on external behaviors, especially aggressive and impulsive behaviors (APA 1994). The current criteria for ASPD, as described in DSM–IV, include a behavioral pattern that begins before age 15 and comprises at least three of the following behaviors:

- Repeated criminal acts
- Deceitfulness
- Impulsiveness
- Repeated fights or assaults
- Disregard for the safety of others
- Irresponsibility
- Lack of remorse.

Because a diagnosis of ASPD according to the DSM–IV criteria requires the presence of only three of the aforementioned behaviors, considerable variability (i.e., heterogeneity) exists among people diagnosed with the disorder. Thus, some ASPD patients may be nonviolent, whereas others may be extremely violent. Some evidence suggests that ASPD patients with histories of violence beginning in childhood may differ from other ASPD patients with respect to certain biological and behavioral characteristics. (This evidence is discussed in more detail in the section Potential Mechanisms Contributing to Alcohol-Related Aggression, p. 9.) Accordingly, clinicians and scientists may find it helpful to study ASPD patients who exhibit repeated violent behaviors as a separate group from other ASPD patients.

ASPD is a relatively common disorder; in fact, it is more common than many other psychiatric disorders, such as bipolar, or manic-depressive, disorder (APA 1994). Overall, approximately 3 percent of men and 1 percent of women in the general population meet the criteria for ASPD (APA 1994). Perhaps not surprisingly, the prevalence of the disorder is even higher in selected populations, such as people in prisons (who include many violent offenders) (Hare 1983). Similarly, the prevalence of ASPD is higher among patients in alcohol or other drug (AOD) abuse treatment programs than in the general population (Hare 1983), suggesting a link between ASPD and AOD abuse and dependence. Accordingly, when investigating the association between alcohol and violence, studying people with ASPD is particularly appropriate.

## Epidemiologic Studies of Alcohol and Violence

Research on the epidemiology of violence has consistently linked alcohol intoxication and violence (Murdoch et al. 1990). For example, a positive correlation exists between the quantity of alcohol consumed and the frequency of a wide variety of violent acts, including sexual assault, child abuse, and homicide. (For more information on this correlation, see other articles in this issue.)

Epidemiologic studies also have noted an association between ASPD and alcohol abuse and dependence. For example, in the Epidemiologic Catchment Area Survey, which surveyed 20,291 persons living at 5 sites across the United States, the people who met the DSM criteria for ASPD were 21 times more likely to develop alcohol abuse and dependence at some point during their lives than were the people who did not have ASPD (Regier et al. 1990).

Some of the behaviors included in the criteria for ASPD (e.g., repeated criminal acts, including driving while intoxicated) could result from alcohol dependence rather than be symptoms of ASPD. To exclude this possibility when examining the relationship between ASPD and problem drinking, researchers have specifically studied people who met the criteria for ASPD before they developed drinking problems. For example, Schuckit (1985) assessed the relationship between ASPD and drinking behaviors in 577 people who entered an alcoholism treatment program. The study found that those people who had ASPD before they developed drinking problems consumed significantly more drinks per day and experienced significantly more alcohol-related problems (e.g., being fired or demoted or spending time in jail) compared with people who did not meet the criteria for ASPD. When analyzed together, these findings suggest that (1) people with ASPD experience higher rates of alcohol abuse and dependence than the general population and (2) people with ASPD who drink to excess are more likely to experience alcohol-related problems than other alcoholics.

As previously discussed, at least some people with ASPD exhibit violent behavior. Without controlled laboratory studies, however, researchers cannot determine whether people with ASPD are more susceptible to alcohol-related aggression than other people, because confounding variables may affect epidemiologic studies on alcohol-related aggression. For example, the positive correlation between the amount of alcohol consumed and increased involvement in acts of violence holds for both perpetrators and victims of violent crimes (Murdoch et al. 1990).

## Laboratory Studies of Aggressive Behavior

Laboratory studies (i.e., studies that use laboratory measures of human aggression) can control for many of the confounding variables that affect epidemiologic studies, thereby allowing for a direct measurement of the association between alcohol and aggression. In these studies, the participant typically is paired with a fictitious opponent with whom the participant competes in performing a certain task. To win the competition, the participant can subject the opponent to actions that most people would consider unwanted or unpleasant (e.g., taking away money from the opponent or exposing the opponent to an electric shock or a loud noise). The quantity or intensity of the unpleasant act performed on the opponent serves as the measure of the participant’s level of aggression.

One widely used example of a laboratory measure of aggression is the Point Subtraction Aggression Paradigm^©^ (Cherek 1992). In this experimental design, each study participant sits in front of a computer monitor and a mechanical box that has two buttons on it. The researchers tell the participants that they must press one of the buttons on their mechanical box as fast as possible to earn money, the amount of which is displayed on their computer screen. The subjects are also told that they each have been paired with another person, or “opponent” (who is actually fictitious). The so-called opponents can ostensibly take money away from the subjects through a computer connection. In turn, the subjects can press the second button on their boxes in order to retaliate and take money from the paired opponents. Periodically, the researchers provoke the participants by having money taken from their accounts, ostensibly by the opponents with whom they are paired. The number of times a participant retaliates by pressing the second button in order to take money from his or her opponent serves as a measure of the participant’s level of aggression.

Although some researchers have argued that these types of laboratory measures are artificial and too far removed from ordinary experience (Tedeschi and Quigley 1996), numerous studies have established the validity of this approach in distinguishing between violent and less violent people, as follows:

- Male parolees convicted of violent felonies exhibited more aggressive responses in the Point Subtraction Aggression Paradigm than did parolees convicted of nonviolent felonies (Cherek et al. 1996, 1997).
- Female parolees with self-reported histories of violent behavior demonstrated significantly more aggressive responses than did female parolees with nonviolent histories (Cherek et al. 2000).
- Although the relationship between a person’s testosterone hormone level and level of aggression is complex, many studies have found an association between increased testosterone levels and high aggression (Archer 1991). Human laboratory studies have confirmed that higher levels of testosterone, whether resulting from testosterone administration (Kouri et al. 1995) or from a person’s own (i.e., endogenous) testosterone levels (Dougherty et al. 1997), were related to higher rates of aggression in both men and women.

In addition to their validity, these laboratory measures allow researchers to study aggression in a controlled environment. Therefore, this approach also has been used to investigate the association between alcohol and aggression.

### Laboratory Studies of Alcohol and Aggression

Bushman and Cooper (1990) reviewed the results of 30 laboratory studies that used the Point Subtraction Aggression Paradigm and other laboratory measures of aggression to assess the effects of alcohol consumption on aggression. Among those studies, several investigations using a variety of laboratory measures of aggression demonstrated that the consumption of alcohol-containing beverages resulted in greater aggressive behavior among the participants than did the consumption of non-alcohol-containing beverages. Based on those studies, the authors concluded that alcohol does in fact increase aggression in humans.

However, high variability exists in the extent to which alcohol is related with aggression as measured in such laboratory studies. Furthermore, as in “real-life” alcohol consumption, not all study participants show increased aggression after drinking alcohol. This variability likely results from individual personality traits that influence a person’s predisposition to aggressive behavior (e.g., the presence of ASPD) both in the presence and absence of alcohol. For example, as mentioned earlier in this section, laboratory studies in which the participants received no alcohol demonstrated that people with histories of behaviors associated with ASPD were more aggressive than were people without such histories. Similarly, male prisoners incarcerated for violent crimes demonstrated more aggressive behavior in laboratory tests than did age-matched college students (Wolfe and Baron 1971).^1^

The influence of a history of aggressive behavior also was demonstrated in a study of cocaine-dependent people who had stopped using cocaine within 2 weeks of participating in the study. In this study Moeller and colleagues (1997) measured aggression using the Point Subtraction Aggression Paradigm and recorded the participants’ self-reported quantity of cocaine used, duration of abstinence from cocaine, severity of withdrawal symptoms, and history of aggressive behavior. The study found that a history of aggression beginning in childhood was the most important predictor of current aggressive behavior in the participants, whereas the amount of cocaine used was a less important predictor. Furthermore, no significant relationship existed between the amount of cocaine withdrawal symptoms and the level of aggressive behavior exhibited by the study participants. These findings support the belief that a life-long history of aggressive behavior (which is one of the criteria for ASPD) plays a critical role in predicting current aggressive behavior.

The importance of various individual differences, including personality characteristics, in determining the susceptibility to aggressive behavior also has been demonstrated in other non-alcohol-related laboratory aggression studies, as follows:

- Women who reported moderate to severe menstrual symptoms were more aggressive across their cycles than women who reported minimal symptoms (Dougherty et al. 1998).
- Men with high levels of trait hostility as measured by hostility-assessing questionnaires showed enhanced susceptibility to increases in aggression after receiving a treatment that lowers the levels of the brain chemical serotonin^2^ (Dougherty et al. 2000).
- Women with borderline personality disorder^3^ demonstrated more aggressive responses than did women without the disorder (Dougherty et al. 1999*a*).

Several laboratory studies have supported the idea that the level of alcohol-related aggression is related to whether a person has a history of past aggressive behavior. For example, Giancola and Zeichner (1995) demonstrated in a study using laboratory measures of aggression that aggressive personality characteristics are associated with alcohol-related aggression. Bailey and Taylor (1991) also demonstrated the interactions between personality traits, alcohol consumption, and aggression. In that study, college students with higher self-reported trait hostility demonstrated more rapid increases in aggression in response to provocation after alcohol consumption than did students with lower trait hostility.

**Figure 1:**
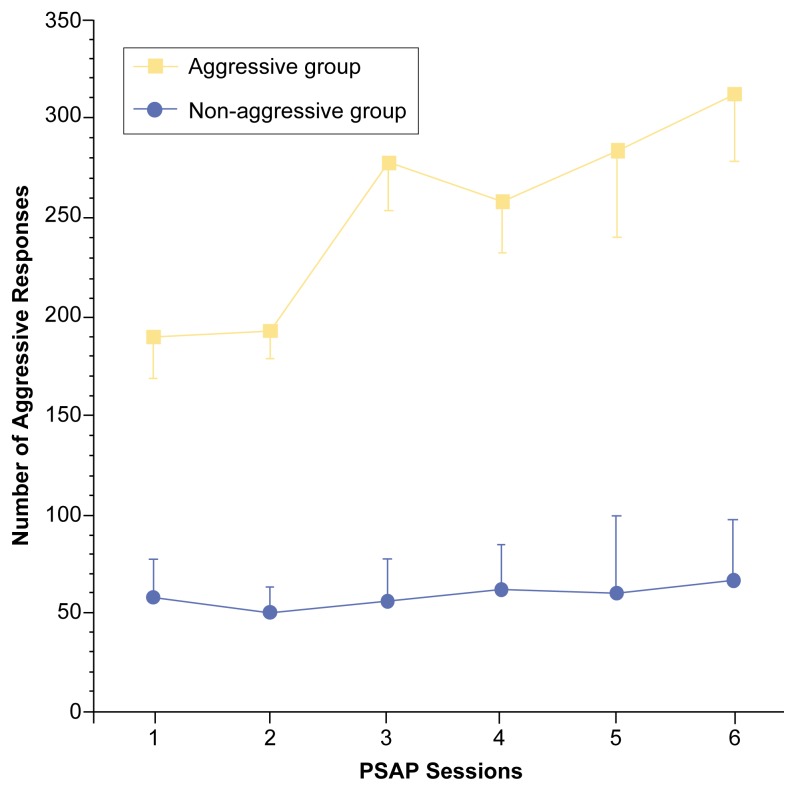
Effects of alcohol consumption on mean number of aggressive responses as determined using the Point Subtraction Aggression Paradigm (PSAP) (Cherek 1992). The participants completed six sessions on each of two test days (i.e., a placebo day and an alcohol dose day). On both days, the participants consumed a beverage 15 minutes before sessions 2, 3, and 4. On the alcohol dose day, each beverage contained alcohol corresponding to approximately 1.5 standard drinks*. The participants were divided into two groups based on high and low aggressive performance on the placebo day. Alcohol ingestion increased aggressive responding in the aggressive group (i.e., participants who had been more aggressive on the placebo day) but not in the nonaggressive group. *A standard drink is defined as one 12-ounce beer, one 5-ounce glass of wine, or 1.5 ounces of distilled spirits.

In a study comparing the effects of alcohol on aggression in both men and women, Dougherty and colleagues (1999*b*) also found evidence for the influence of individual differences in personality characteristics. In that study, both the men and women exhibited similar increases in aggression after consuming alcohol. Further analysis indicated, however, that these increases in aggression were particularly high in the participants who demonstrated elevated levels of “aggressive responding” even when they did not receive alcohol (i.e., under placebo conditions). In other words, participants with the strongest tendencies for aggression while sober exhibited the greatest increases in aggression after consuming alcohol. Of particular interest, the proportion of men and women in the low- and high-aggression groups was nearly equal (see figure 1).

The observations described in the previous paragraph suggest that people with ASPD, many of whom have particularly strong aggressive tendencies, should be especially prone to alcohol-related aggression. To explore this notion in more detail, Moeller and colleagues (1998) compared alcohol’s effect on aggression in 8 people who met the criteria for ASPD with 10 people who did not meet those criteria. As was to be expected based on the diagnostic criteria, the participants with ASPD had committed significantly more aggressive acts over their lifetimes than had the non-ASPD participants.

**Figure 2:**
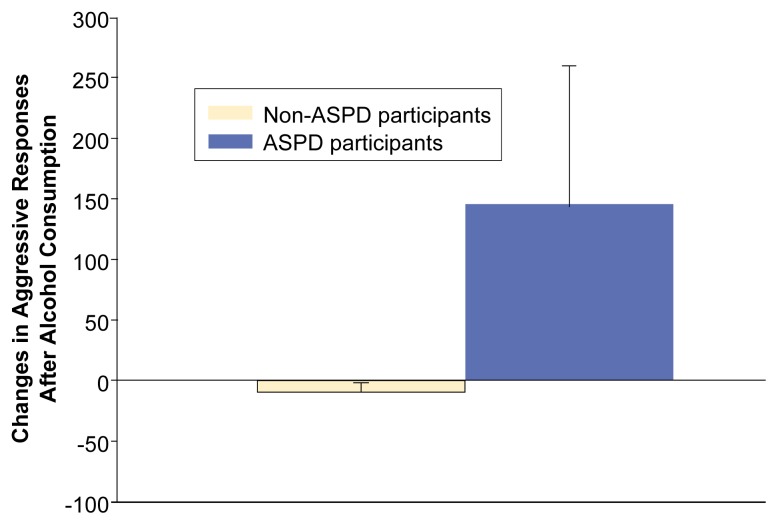
Alcohol’s effect on aggressive responding in both people with and people without antisocial personality disorder (ASPD). Researchers compared the levels of aggressive responding in study participants after they consumed a nonalcoholic beverage (i.e., placebo) with responses after they consumed 1 gram of alcohol per kilogram body weight (corresponding to approximately four standard drinks*). In people without ASPD, alcohol consumption slightly decreased aggressive responding, whereas in people with ASPD, alcohol consumption substantially increased aggressive responding. *A standard drink is defined as one 12-ounce beer, one 5-ounce glass of wine, or 1.5 ounces of distilled spirits.

During the study, the participants each received four drinks (one drink per day) containing varying amounts of alcohol before their level of aggression was assessed using the Point Subtraction Aggression Paradigm. The study found a significant difference in alcohol’s effect on aggressive responding between participants with and without ASPD. Thus, participants with ASPD exhibited a greater increase in aggressive responding after consuming alcohol than did the non-ASPD participants (see figure 2). As with previous research using other samples, the alcohol-related increase in aggressive behavior was positively correlated with the number of past aggressive acts the participants had committed.

When analyzed together, the findings of these human laboratory studies on the association between aggression, personality characteristics, and alcohol consumption allow three main conclusions, as follows:

- As a group, people with ASPD appear to be more aggressive than people without ASPD, although not all people with ASPD show increased aggressive behavior.
- The most important predictor of current aggressive behavior appears to be the amount of aggressive behavior a person has demonstrated in the past.
- People who are more likely to be aggressive when sober, regardless of the underlying reasons, are more likely to exhibit increased aggressive behavior when under the influence of alcohol.

## Potential Mechanisms Contributing to Alcohol-Related Aggression

Several theories exist regarding the causes underlying alcohol-related aggression. Some of these theories focus on the roles of expectancies, changes in brain function, and changes in brain chemistry.

### Expectancies

The term “expectancies” refers to the notion that a person has certain assumptions about alcohol’s effects on behavior. For example, common expectancies associated with alcohol consumption are that alcohol will make the drinker less inhibited and more outgoing. Such expectancies can influence a drinker’s behavior, because people are more likely to behave under the influence of alcohol in the manner in which they expect to behave.

Evidence for the effect of expectancies derives from studies in which participants were first asked to describe the way they believed alcohol influenced behavior. Subsequently, the participants received a drink that they were told contained alcohol, but which in fact did not contain significant amounts of alcohol. The study found that the participants showed increases in those behaviors that they believed would be increased by alcohol, even though they had not received any alcohol (Leigh 1989). Thus, the observation that people with a history of aggressive behavior show increased aggression under the influence of alcohol may result from the fact that these people expect alcohol to make them more aggressive.

Expectancies probably are not the sole explanation for alcohol-related aggression, however. In fact, to reduce the influence of expectancies, researchers conducting studies using laboratory measures of aggression generally take great care not to mention the study’s purpose to the participants. For example, in the study by Moeller and colleagues (1998) on the association between ASPD and alcohol-related aggression, the participants were told that the study’s purpose was to measure alcohol’s effects on mood and reaction time. Furthermore, in another study demonstrating that alcohol increased aggression significantly more than an active placebo,^4^ Chermack and Taylor (1995) detected no significant effect of expectancies on behavior and no significant interaction between expectancies and the alcohol dose used.

### Changes in Brain Function

Another theory regarding the mechanisms through which alcohol increases aggression centers around the way that alcohol “disinhibits,” or brings out, behaviors that are normally repressed. This theory is based on the concept that alcohol impairs higher reasoning, or “executive,” brain functions, allowing more basic or impulsive brain functions to take over. Support for this theory derives from study findings indicating that alcohol intoxication impairs several aspects of higher brain functioning, including planning, verbal fluency, attention, and memory (Peterson et al. 1990; Dougherty et al. 1999*c*). Likewise, some studies have found that people with ASPD experience deficits in higher reasoning or executive cognitive brain function (Giancola and Moss 1998), thereby suggesting that these people may be more susceptible to alcohol’s aggression-inducing effects.

### Changes in Brain Chemistry

Alcohol has direct biochemical effects on the brain itself. Although alcohol’s effects on the brain are complex and not fully understood, researchers have demonstrated that alcohol alters the activities of several brain chemicals (i.e., neurotransmitters), including γ-aminobutyric acid (GABA) and serotonin. Both of these neurotransmitters have been associated with aggressive behavior.

GABA is the major inhibitory neurotransmitter in the brain—that is, it depresses the activity of the nerve cells it regulates. Like other neurotransmitters, GABA modulates the activity of a cell by interacting with certain molecules (i.e., receptors) on the cell’s surface, thereby initiating a chain of biochemical reactions that result in altered nerve cell activity. Two main types of GABA receptors exist, GABA_A_ and GABA_B_. Alcohol, like certain sedating agents (e.g., benzodiazepines, such as Valium^®^, and barbiturates), enhances GABA’s effect on the GABA_A_ receptor. Thus, at least some of alcohol’s effects probably are mediated through this neurotransmitter in select brain regions (Cooper et al. 1991). Furthermore, animal studies have found that treating animals with medications which block GABA activity can diminish alcohol-related aggression (Miczek et al. 1993). Thus, alcohol may increase aggression, at least in part, by directly affecting GABA activity.

Alcohol also affects serotonin levels in the brain. Serotonin is a neurotransmitter that is widely distributed in the brain. It is involved in the coordination of complex motor and sensory patterns of behavior, such as sleep, appetite, and mood (Cooper et al. 1991). Most studies have found that alcohol increases brain serotonin levels, although other studies have noted that alcohol decreases serotonin levels, at least in some brain regions (LeMarquand et al. 1994). Alcohol’s effects on brain serotonin may contribute to alcohol-related aggression, because studies of impulsively violent people with ASPD have found that those people have lower serotonin levels than do nonviolent people (Brown et al. 1979). Consequently, alcohol-related reductions in serotonin levels may exacerbate the susceptibility to aggressive behavior in people with ASPD. (For more information on serotonin’s role in alcohol-related aggression, see the article in this issue by Higley, pp. 12–19).

## Conclusions

People with ASPD are more likely to meet the criteria for alcohol abuse or dependence and, as discussed in this article, are more susceptible to alcohol’s aggression-related effects than people without the disorder. Several studies have demonstrated that aggressive personality traits are associated with an increase in aggression after drinking alcohol. Furthermore, in a study of people with ASPD, those participants who had exhibited the most aggression in the past were most likely to become aggressive under the influence of alcohol. Researchers have developed several theories to explain the cause of alcohol-related aggression that focus on expectancies, brain function, and brain chemistry. However, it is unlikely that any one factor can sufficiently explain the association between alcohol consumption and increased aggression.

Human laboratory studies have led to significant advances in understanding the relationship between personality and alcohol-related aggression. Several questions remain, however, regarding the role of ASPD in this relationship. For example, it is still unclear whether the association of alcohol-related aggression with ASPD results from some key feature of ASPD itself or from the difficulties that many people with ASPD have in controlling aggressive or impulsive behaviors. Similarly, researchers have not yet determined the neurological and biochemical factors underlying alcohol-related aggression. Once scientists have further elucidated these issues, researchers may be able to develop successful treatment approaches aimed at decreasing alcohol-related aggression. Because alcohol-related violence continues to be a significant public health problem, further research on the relationship between ASPD and alcohol-related aggression clearly is warranted.

## Footnotes

1 Although these prisoners were not diagnosed with ASPD according to the current criteria, some criteria of ASPD, such as impulsive and aggressive behavior, are highly likely to be present in persons incarcerated for violent crimes.

2 Low levels of serotonin have been associated with certain personality characteristics, such as aggression and depression.

3 Borderline personality disorder, which occurs primarily in women, is characterized by a pattern of intense and self-destructive relationships along with impulsive behaviors (APA 1994).

4 An active placebo in this case is a drink that contains enough alcohol to have an alcoholic aroma but not enough alcohol to result in detectable blood alcohol levels.
